# Uropygial gland and bib colouration in the house sparrow

**DOI:** 10.7717/peerj.2102

**Published:** 2016-06-02

**Authors:** Gregorio Moreno-Rueda

**Affiliations:** Departamento de Zoología, Universidad de Granada, Granada, Spain

**Keywords:** LPS, House sparrow, Mate choice, Cosmetic, *Passer domesticus*, Uropygial gland, Sexual selection

## Abstract

Birds frequently signal different qualities by plumage colouration, mainly during mating. However, plumage colouration is determined during the moult, and therefore it would indicate the quality of individual birds during the moult, not its current quality. Recent studies, however, suggest that birds could modify plumage colouration by using cosmetic preen oil produced by the uropygial gland. In this study, I show that bib colouration is related to uropygial gland size and body condition in male house sparrows (*Passer domesticus*). Moreover, I conducted an experiment in which a group of sparrows were inoculated with an antigen, mimicking an illness. In control birds, short-term changes in bib colouration were related to both body condition and change in uropygial gland size. Therefore, birds that reduced uropygial gland size showed a greater colouration change. However, bib colouration did not change with the change in uropygial gland size in experimental birds inoculated with the antigen. Given that the experiment did not affect preen oil production or consumption, this finding tentatively suggests that the immune challenge provoked a change in the composition of preen oil, affecting its cosmetic properties. In short, the results of this study suggest that (1) male house sparrows produce cosmetic preen oil that alters the colouration of their bibs; (2) the more change in uropygial gland size, the more change in bib colouration; and (3) in this way, bib colouration has the potential to signal current health status, since less healthy birds showed less capacity to change bib colouration.

## Introduction

Animals frequently use patches of colouration in communication (Bradbury & Vehrencamp, 2011). Bird plumage colouration, in particular, is a recurrent model system for the study of communication based on colouration (Hill & Mcgraw, 2006). Plumage colouration is produced by pigments embedded in the feathers, as well as by the structure of keratin layers (Hill & Mcgraw, 2006). In order for communication to be useful (for the receiver), colour patches should convey some type of information about the determinate quality of the bearer (Searcy & Nowicki, 2005). In this sense, signals based on plumage colouration have the problem that coloured plumage patches are formed during the moult and thus presumably reflect the bearer condition (for a given quality) during the moult (e.g., Vágási, Pap & Barta, 2010), not the current quality status. Although the current bearer quality status might be correlated with that during the moult (Saks, Ots & Hõrak, 2003), this may not be necessarily true. For example, if the patch colour signals health status during the moult and the population suffers an epidemic after the moult was completed, the signal would become uninformative.

Nevertheless, plumage colouration is not invariable and indeed changes with time (e.g., Örnborg et al., 2002; Figuerola & Senar, 2005; Delhey et al., 2006), primarily by physical abrasion. Moreover, plumage colouration may change from the addition of cosmetics (reviews in Montgomerie, 2006b; Delhey, Peters & Kempenaers, 2007). One of the main cosmetics used by birds is the secretion of uropygial gland (hereafter, preen oil), an oily secretion that birds spread on their plumage during preening (Clark, 2004). Several studies show that preen oil effectively alters plumage colouration (Surmacki & Nowakowski, 2007; López-Rull, Pagán, & García, 2010; Amat et al., 2011; Pérez-Rodríguez, Mougeot & Bortolotti, 2011; but see Delhey et al., 2008). Indeed, it has been proposed that, by changing plumage colouration with the addition of preen oil, birds may “update” the information contained in the signal about the quality status of the sender (Montgomerie, 2006b; López-Rull, Pagán, & García, 2010). However, although it is well established that preen oil changes plumage colouration, it is unknown whether it does it in a way that in fact updates the message contained in the signal, so that the new colouration indicates the current condition of the bearer, instead of the condition when it moulted. The only evidence in favour of this hypothesis comes from the beak colour of tawny owlets (*Strix aluco*). In these nestlings, beak brightness is influenced by preen oil, and when an infection is mimicked by the inoculation of an antigen (lipopolysaccharide from the cell wall of *Escherichia coli*, LPS), the secretion of preen oil is reduced and consequently the beak becomes lighter (Piault et al., 2008). However, it is unknown whether beak colouration is used as a signal in these chicks (perhaps signalling health to their parents), or the colour change detected was simply a by-product of using the beak to smear the preen oil on the plumage.

A recent study has shown that an immune challenge reduces preen-oil production in the house sparrow (*Passer domesticus*) in a body-condition-dependent fashion (Moreno-Rueda, 2015), similarly to tawny owlets (see Piault et al., 2008). Thus, these studies suggest that preen-oil production is influenced by health status (also see Pap et al., 2013). Consequently, I hypothesise that (1) the bib colour of male house sparrows may be modified by preen oil, (2) house sparrows facing an immunological challenge may find their preen oil production impaired, which in turn (3) will affect the colouration of their bibs, and in this way (4) bib colouration may indicate current health status of males. The size of black bib of house sparrows intervenes in intraspecific communication (Anderson, 2006; Nakagawa et al., 2007), both in mate choice (Møller, 1988; Griggio & Hoi, 2010; but see Kimball, 1996) and signalling dominance status (Møller, 1987; González et al., 2002; McGraw, Dale & Mackillop, 2003). Most studies have focussed on bib size (Nakagawa et al., 2007), but bib lightness and saturation are strongly correlated (negatively and positively, respectively) with bib size (Václav, 2006). Moreover, bib size is positively related to immunocompetence (Møller, Kimball & Erritzøe, 1996; González, Sorci & De Lope, 1999).

In order to test the hypothesis that bib colouration is affected by immunocompetence in a way mediated by preen oil production, I examined the relationship between bib colouration and uropygial gland size, and carried out an experiment in which a group of house sparrows were inoculated with LPS, while a second group served as control (these birds were sham-inoculated with phosphate-buffered saline, PBS). Subsequently, I examined changes in bib colouration as a consequence of the experiment.

## Methods

### General procedure

The study was conducted during March 2011 with 21 adult male house sparrows captured with mist-nets on a farm in Padul (SE Spain, 37°01′N, 3°37′W) and transported to an outdoor aviary located in Moraleda de Zafayona (37°11′N, 3°57′W). No bird suffered any damage during capture, transport, maintenance in the aviary, or as a consequence of the experiment. The aviary structure followed the recommendations of the European directive as well as national legislation. Measuring about 20 m^3^, the aviary was built with bricks at the base (1-m height) and a complete wall, the remaining being covered with a mesh supported by a metal framework. The structure was designed to avoid injuring the birds. A roof was provided to protect birds from rainfall and direct sunlight. All birds were individually marked with colour rings, and were supplied with food (seed mixture, fruit, and different vitamins and minerals) and water with *ad libitum* access, as well as diverse perches, and trays with water and powder for bathing and dust bathing. The aviary, and especially the food and water containers, were carefully cleaned and disinfected before the capture of the birds. Confinement lasted for a week, and when the study ended, the sparrows were released in the same place where they had been captured. The study was performed with the permission of the Andalusian government.

On 03 March 2011, 12 sparrows were subcutaneously injected in the patagium with 0.1 mg of LPS (serotype 055:B5, L-2880, Sigma Aldrich), diluted in 0.01 ml of isotonic phosphate-buffered saline. LPS acts as an antigen, provoking a humoral immune reaction that mimics an infection, and diverts energy from other functions to the immune system. Consequently, inoculating LPS usually lowers body mass in the house sparrow (Bonneaud et al., 2003; Moreno-Rueda, 2011). Another nine sparrows were injected with 0.01 ml of PBS as a sham control. To determine whether the antigen effectively stimulated the immune system, I measured the thickness of the patagium where the substances were inoculated with a pressure-sensitive micrometre (Mitutoyo; accuracy 0.01 mm). Measurements were taken before injecting the substance and four hours afterwards, when an immune response to LPS is already detectable (Parmentier, De Vries Reilingh & Nieul, 1998). Then, I tested whether the patagium was significantly swelled in LPS-inoculated birds, which indicates an immune response to the antigen.

I took a number of measurements just prior the experiment and just when the experiment was ended (on 10 March). Firstly, I measured the length, width, and height (from the base of the gland to the base of the papilla) of the uropygial gland (three times each) with a digital calliper (accuracy 0.01 mm), and estimated its size by multiplying the three measurements, which is a good estimator of gland volume and preen-oil production in house sparrows (Pap et al., 2010). I estimated the repeatability of uropygial gland size by measuring twice the gland in 21 individuals randomly selected (following Lessells & Boag, 1987). Repeatability was 0.76 (*F*_1,19_ = 72.7, *p* < 0.001). Also, I measured body mass with a digital balance (accuracy 0.1 g), and wing length with a ruler (accuracy 0.5 mm). Body condition was estimated as the residuals of the regression of body mass (log-transformed) against the wing length (log-transformed) as a measurement of skeletal body size (review in Green, 2001). Furthermore, I measured bib size by means of photography on gridded paper with a digital camera (Fujifilm 10.2 megapixels; following Figuerola & Senar, 2000). The camera was mounted on a tripod, consistently at the same distance from birds, which were held with the breast plumage combed in order to ensure a normal position. Afterwards, patch surface areas were measured with the program Image J (Abramoff, Magelhaes & Ram, 2004). Photos were scaled using the gridded paper as reference. Then, I adjusted the area using the “colour threshold” tool, and measured the area of bib of each bird with the “analyse particles” tool.

Plus, the colouration of the bib was measured with a spectrophotometer (Minolta CM-2600d). After reference calibration of white, the spectrophotometer was placed over the bib and three beams of light were projected through a hole of 3 mm in diameter. As a result, three reflectance measurements were taken and automatically averaged (Andersson & Prager, 2006). The spectrophotometer did not measure the ultraviolet spectrum, whose measurement is unnecessary given that house sparrows do not reflect ultraviolet radiation in the bib (Václav, 2006). Bib colouration was measured in the L*a*b* colour-space of the *Commission Internationale d’Eclairage* (CIE; Montgomerie, 2006a). L*a*b* is a three-dimensional rectangular colour space. L* axis represents lightness (0 is completely black, 100 is completely white); a* axis represents red-green gradient (positive values are red, negative values are green); b* axis represents blue-yellow gradient (positive values are yellow, negative values are blue). From L*a*b* values I determined the saturation, i.e., the radiance in a specific part of the spectrum in relation to the radiance from the whole visible spectrum. Saturation was calculated as C* = [(a*)^2^ +(b*)^2^]^1∕2^, measured as the percentage distance from the centre of the colour space to its circumference, where pure spectral colours are represented. Hue angle (the “colour” in common parlance) was calculated as H* = tan^−1^(b*/a*) (Endler, 1990).

### Statistical analyses

In a first analysis, with data taken prior the inoculation of LPS and PBS, I used the *t*-test to check for differences between LPS- and PBS-inoculated sparrows for wing length (as indicator of structural body size), body condition, uropygial gland size, bib size, and bib colouration (bib lightness, saturation, and hue). In addition, I examined the relationships (by running Pearson correlations) among the different biometrical variables (bib size, wing length, body condition, and uropygial gland size) and bib colouration parameters (lightness, saturation, and hue). I corrected for multiple tests by applying sequential Bonferroni (Rice, 1989), but, given that the correction of Bonferroni increases the rate of statistical error type II (Moran, 2003), I show the uncorrected *P*-values, indicating if they remain significant when corrected. Given that bib saturation was correlated with body condition, uropygial gland size, and bib size (see ‘Results’), in order to ascertain the independent relationships among these variables with bib saturation, I carried out a multiple regression model (Linear Model) of type-III Sums of Squares (Quinn & Keough, 2002). Collinearity among continuous variables was checked by examining tolerance, which was always >0.90, indicating that collinearity was consistently very low (Quinn & Keough, 2002). Normality and homoscedasticity of residuals of the models were checked according to Shapiro–Wilks and Levene’s tests, respectively (Quinn & Keough, 2002), and, when necessary, the variables were log-transformed in order to improve the fit of the models. Similar multiple regression models with bib lightness and hue as dependent variables were not carried out (as unnecessary) because these variables did not correlate with more than one biometrical variable (see below).

In order to confirm that the inoculation of LPS effectively produced an immune response, I tested with paired *t*-test for differences in patagium swelling before and 4-h after the inoculation in both LPS- and PBS-inoculated sparrows. I estimated the change in patagium swelling (final minus initial patagium swelling) and used a *t*-test for unpaired samples to examine for differences in change in patagium swelling between treatments. Also, I used the *t*-test to check for differences between treatments in the changes (final value minus initial value) in uropygial gland size, body condition, and bib colouration (lightness, saturation, and hue), measured once the experiment ended (seven days after inoculations). Paired *t*-test were also used to examine the within-individuals change in uropygial gland size.

Lastly, in order to examine which variables were related to changes in bib colouration during the experiment, I carried out correlations among the change in bib colouration variables (change in lightness, saturation, and hue) and bib size, body condition, and both the change in uropygial gland size and in body condition. I identified changes in colouration correlated with initial body condition and change in uropygial gland size (but not with bib size or change in body condition; see ‘Results’). Therefore, in order to ascertain the independent effect of treatment, body condition, and change in uropygial gland size, I performed a set of Linear Models with the change in every component of bib colouration (lightness, saturation, and hue) as dependent variables, and body condition, change in uropygial gland size, treatment, and all the interactions among these variables as predictors. Moreover, although both bib size and change in body condition were uncorrelated with changes in colouration, in order to be sure that these variables did not bias the results, I repeated the models including these variables as predictors. Non-significant interactions (for which *P* > 0.05) were removed from models (following Engqvist, 2005). Moreover, models including all interactions did not significantly differ from models including only significant interactions (*F*-ratio for comparing models, in all cases *P* > 0.25). As described above, normality and homoscedasticity of residuals of the models were checked according to Shapiro–Wilks and Levene’s tests, respectively (Quinn & Keough, 2002), and, when necessary, the variables were log-transformed in order to improve the fit of the models.

The complete dataset is available in Table S1. All analyses were performed with STASTISTICA 8.0 (StatSoft, Tulsa, OK, USA).

## Results

### Descriptive relationships among variables prior the experiment

Prior to the experiment, no statistical differences were found between control (PBS-inoculated) and experimental (LPS-inoculated) house sparrows in wing length, body condition, uropygial gland size, and bib colouration (light, saturation, and hue) (Table 1). However, despite the randomization of the treatment assignment, bib size was significantly larger in PBS- than in LPS-inoculated birds (Table 1). Nevertheless, differences in bib size turned not significant when applying Bonferroni correction and, moreover, this possible differences did not seem to affect results of the experiment (see analyses below and Table 3C). Bib size was not significantly correlated with any colour parameter (Table 2), although there was an almost significant trend for sparrows with larger bibs to have more saturated bibs (*r* = 0.416, *P* = 0.061). Bib saturation, moreover, was negatively correlated with body condition, and uropygial gland size (Table 2). Notice that uropygial gland size was not correlated with body condition (*r* = 0.17, *P* = 0.45) or bib size (*r* = − 0.10, *P* = 0.66), and body condition was not correlated with bib size (*r* = 0.18, *P* = 0.43). When body condition, uropygial gland size, and bib size were included as predictors in a multiple regression model, a significant correlation between bib size and bib saturation emerged (partial *r* = 0.47, *P* < 0.01), meanwhile bib saturation remained significantly correlated with body condition (partial *r* = − 0.53, *P* < 0.01), and uropygial gland size (partial *r* = − 0.40, *P* = 0.01) (multiple *R* = 0.83, *F*_3,17_ = 12.46, *P* < 0.001, adjusted *R*^2^ = 0.63, tolerance > 0.92). On the other hand, bib hue was also positively correlated with uropygial gland size, but bib lightness was not correlated with any variable (Table 2). Saturation and hue were significantly correlated among themselves (*r* = 0.664, *P* = 0.001), but not with lightness (in both cases, *r* < 0.4, *P* > 0.10).

**Table 1:** Means (with SD) of variables measured between control (PBS-inoculated) and experimental (LPS-inoculated) sparrows, and the result of the test for the differences. Raw values were used to calculate the means, but variables were transformed when necessary for the *t*-test. Significant differences appear in bold, although no difference remained significant after correction by Bonferroni.

	LPS (*n* = 12)		PBS (*n* = 9)		*t*_19_	*P*
	Mean	SD	Mean	SD
Wing length (mm)	80.58	1.94	80.89	0.86	–0.46	0.651
Initial body condition (residuals)	–0.02	0.04	0.02	0.06	–1.86	0.078
**Bib size (cm^2^)**	**15.42**	**2.71**	**19.27**	**4.45**	**−2.46**	**0.024**
Initial uropygial gland size (mm^3^)	0.10	0.02	0.11	0.03	–1.39	0.181
Initial bib lightness	21.77	5.16	19.14	5.72	1.10	0.284
Initial bib saturation	2.14	0.74	1.92	1.01	0.56	0.584
Initial bib hue	0.57	0.26	0.82	0.53	–1.19	0.251

**Table 2:** Matrix of correlations among the variables measured in house sparrows before the start of the experiment and bib colouration (hue, saturation, and lightness). Significant or almost significant correlations are indicated in bold, although no correlation remained significant when the correction of Bonferroni was applied. * for *P* < 0.05, and § for *P* = 0.061.

	Bib lightness	Bib saturation	Bib hue
Wing length	0.153	–0.345	0.155
Body condition	–0.161	−0.515*	0.257
Uropygial gland size	–0.335	−0.542*	0.453*
Bib size	–0.083	**0.416^§^**	−0.269

**Table 3:** Statistical models examining the causes of the change in bib colour. (A) Results of the linear models examining the effect of treatment, change in uropygial gland size ($\mrm{\Delta }$UGS), body condition, and the interaction treatment × $\mrm{\Delta }$UGS on the change in colour parameters (lightness, saturation, and hue) of bib. Degree of freedom of error term were one less for lightness, as an outlier (Fig. S1) was removed (see Table S2 for the results including the outlier). (B) Linear models examining the effect of treatment, change in body condition (not included in the previous model), $\mrm{\Delta }$UGS, initial body condition, and the interaction treatment × $\mrm{\Delta }$UGS on changes in colour parameters (lightness, saturation, and hue) of bib. (C) Linear models examining the effect of treatment, bib size (not included in models in A and B), $\mrm{\Delta }$UGS, body condition, and the interaction treatment × $\mrm{\Delta }$UGS on colour parameters (lightness, saturation, and hue) of bib. For every effect, standardised coefficients (*β* ± SE), *F*- and *P*-value are indicated. Significant results (*P* < 0.05) in bold. For every model, Adjusted *R*^2^ and AIC is indicated.

(A) Model excluding change in body condition and bib size									
	Change bib lightness Adj. *R*^2^ = 0.11; AIC = 152.0			Change bib saturation Adj. *R*^2^ = 0.53; AIC = 49.1			Change bib hue Adj. *R*^2^ = 0.45; AIC = 21.1		
	*β* ± SE	*F*_1,15_	*P*	*β* ± SE	*F*_1,16_	*P*	*β* ± SE	*F*_1,16_	*P*
Treatment	130 ± 95	1.880	0.191	170 ± 62	7.501	**0.015**	−135 ± 67	4.043	0.062
Body condition	0.33 ± 0.25	1.703	0.212	0.57 ± 0.17	11.530	**0.004**	−0.35 ± 0.18	3.783	0.070
$\mrm{\Delta }$UGS	0.01 ± 0.27	0.001	0.977	−0.15 ± 0.18	0.740	0.402	0.42 ± 0.19	4.774	**0.044**
Treatment*$\mrm{\Delta }$UGS	−130 ± 95	1.875	0.191	−170 ± 62	7.550	**0.014**	135 ± 67	4.052	0.061

(B) Model including change in body condition									
	Change bib lightness Adj. *R*^2^ = 0.05; AIC = 153.9			Change bib saturation Adj. *R*^2^ = 0.50; AIC = 51.1			Change bib hue Adj. *R*^2^ = 0.43; AIC = 22.8		
	*β* ± SE	*F*_1,14_	*P*	*β* ± SE	*F*_1,15_	*P*	*β* ± SE	*F*_1,15_	*P*
Treatment	131 ± 99	1.768	0.205	170 ± 65	6.792	**0.020**	−129 ± 70	3.438	0.083
Change in body condition	−0.03 ± 0.25	0.012	0.915	0.01 ± 0.18	0.003	0.960	−0.09 ± 0.19	0.227	0.641
Body condition	0.32 ± 0.28	1.246	0.283	0.57 ± 0.19	9.473	**0.008**	−0.39 ± 0.20	3.764	0.071
$\mrm{\Delta }$UGS	0.01 ± 0.28	0.001	0.970	−0.16 ± 0.19	4.757	**0.046**	0.44 ± 0.20	4.757	**0.046**
Treatment × $\mrm{\Delta }$UGS	−131 ± 99	1.763	0.205	−170 ± 65	6.840	**0.019**	129 ± 70	3.449	0.083

(C) Model including bib size									
	Change bib lightness Adj. *R*^2^ = 0.05; AIC = 154.0			Change bib saturation Adj. *R*^2^ = 0.57; AIC = 47.7			Change bib hue Adj. *R*^2^ = 0.45; AIC = 21.7		
	*β* ± SE	*F*_1,14_	*P*	*β* ± SE	*F*_1,15_	*P*	*β* ± SE	*F*_1,15_	*P*
Treatment	124 ± 103	1.445	0.249	139 ± 62	5.019	**0.041**	−113 ± 70	2.586	0.129
Bib size	−0.06 ± 0.33	0.038	0.848	−0.35 ± 0.21	2.599	0.128	0.24 ± 0.24	0.997	0.334
Body condition	0.35 ± 0.27	1.596	0.227	0.67 ± 0.17	15.216	**0.001**	−0.42 ± 0.19	4.717	**0.046**
$\mrm{\Delta }$UGS	0.03 ± 0.30	0.009	0.924	−0.03 ± 0.19	0.019	0.891	0.33 ± 0.21	2.437	0.139
Treatment × $\mrm{\Delta }$UGS	−124 ± 103	1.439	0.250	−140 ± 62	5.040	**0.040**	113 ± 70	2.584	0.129

### Effects of the experiment on immune system and uropygial gland size

The experimental treatment had a significant effect on the house sparrows’ immune system. Sparrows in the LPS-inoculated group showed a significant patagium swelling 4 h after the inoculation (average ± SD variation in patagium thickness: 0.37 ± 0.20 mm, *t*_11_ = 6.29, *P* < 0.001), while the control group showed no swelling (change in thickness: −0.03 ± 0.06 mm, *t*_8_ = 1.33, *P* = 0.22), difference in swelling between LPS- and PBS-inoculated sparrows being significant (*t*_19_ = 6.16, *P* < 0.001). For all individuals considered together, the uropygial gland size decreased during the experiment (in average, −0.01 ± 0.02 mm^3^; paired *t*-test, *t*_20_ = 2.77, *P* = 0.011); nevertheless, the change in uropygial gland size did not differ between treatments (*t*_19_ = 1.55, *P* = 0.14).

### Determinants of changes in bib colouration during the experiment

**Figure 1:**
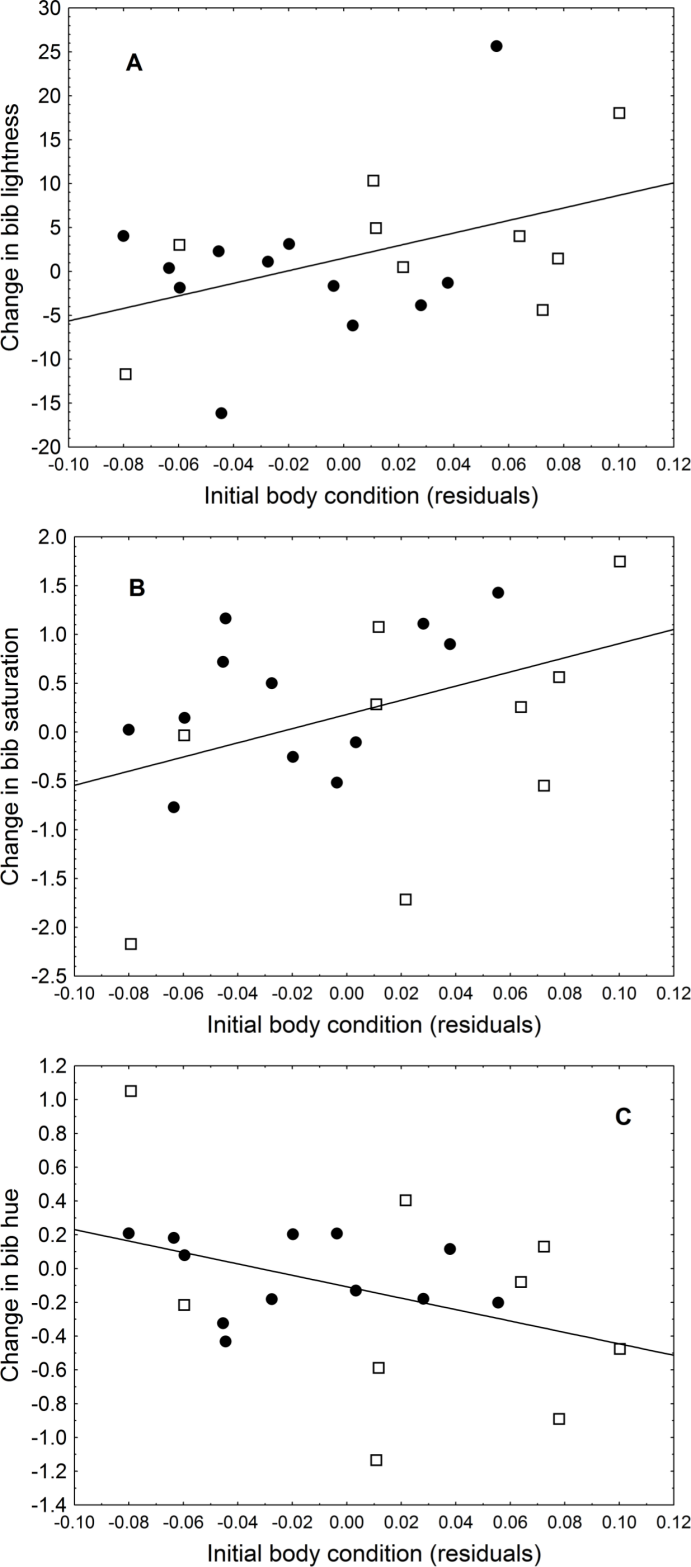
Relationship between initial body condition and (A) change in bib lightness, (B) change in bib saturation, and (C) change in bib hue. Black circles are LPS-inoculated individuals, empty squares are PBS-inoculated sparrows.

The change in uropygial gland size correlated positively with the change in hue (*r* = 0.61, *P* = 0.003). Initial body condition also was related to changes in colouration, as birds in better condition had a greater change in lightness (*r* = 0.44, *P* = 0.046) and trended (almost significantly) to have a greater change in saturation (*r* = 0.41, *P* = 0.066) and hue (*r* = − 0.40, *P* = 0.073) (Fig. 1). Bib size or change in body condition were not related to change in any colouration parameter (in all cases ∣*r*∣ < 0.4, *P* > 0.05).

**Figure 2:**
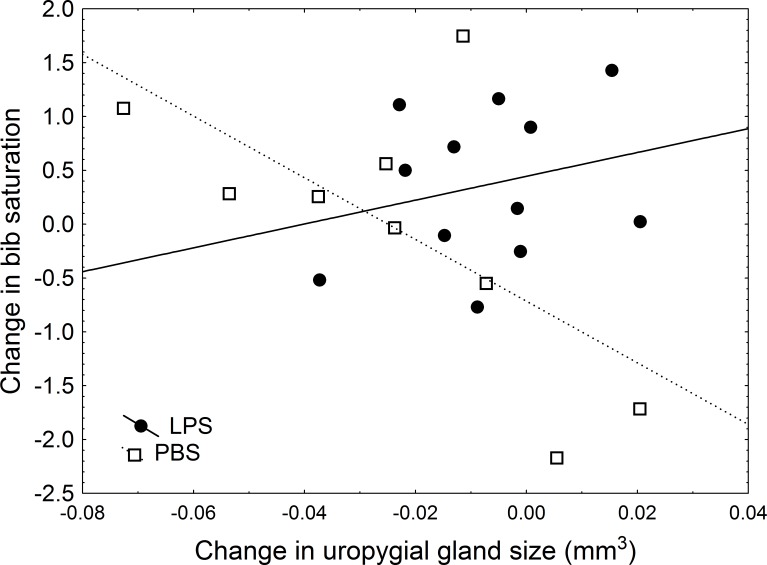
Relationship between change in uropygial gland size and change in bib saturation. Black circles and solid line are LPS-inoculated individuals, empty squares and dashed line are PBS-inoculated sparrows.

In a more detailed analysis, I examined the effect of the treatment, change in uropygial gland size (hereafter, $\mrm{\Delta }$UGS), and body condition on the change in colouration by using Linear Models in which, therefore, the effect of each variable was controlled for the effect of the other variables introduced in the model. After the experiment was carried out, I found a significant effect of body condition, treatment, and the interaction treatment × $\mrm{\Delta }$UGS on change in bib saturation (Table 3). In this model, a significant effect of the treatment emerged: in sparrows inoculated with LPS, bib saturation did not change significantly (average change 0.45, with lower and upper 95% CI limits of −0.004 and 0.91). However, in the control group, the bib became less saturated, with an average change of −0.54 (95% CI limits: −1.06 and −0.026; significantly below zero (Nakagawa & Cuthill, 2007)). Moreover, there was a positive effect of body condition on change in saturation (*β* = 0.568). Regarding the interaction between treatment and change in uropygial gland size, experimental individuals showed no correlation between change in hue and $\mrm{\Delta }$UGS (*r* = 0.25, *P* = 0.43), while in control birds there was an almost significant trend for a greater decrease in uropygial gland size accompanying greater change in saturation (*r* = − 0.66, *P* = 0.053; Fig. 2). These findings remained significant in models in which change in body condition or bib size were included (Tables 3B and 3C).

Change in hue was significantly determined by $\mrm{\Delta }$UGS, with an almost significant effect of treatment, body condition, and the interaction treatment × $\mrm{\Delta }$UGS (Table 3). More specifically, in the control group, there was a significant correlation between $\mrm{\Delta }$UGS and change in hue (*r* = 0.72, *P* = 0.03). By contrast, in the experimental group, such a correlation was inexistent (*r* = 0.11, *P* = 0.73). However, these results were not robust when in the model I corrected for bib size (Table 3C). For the case of lightness, in a first analysis, I found a significant interaction between $\mrm{\Delta }$UGS and treatment (Table S2), but this finding seemed to be caused by an outlier (Fig. S1). When the outlier was removed from the analyses, no significant result emerged (Table 3). Lastly, the change in lightness correlated with change in saturation (*r* = 0.44, *P* = 0.045), but not with the change in hue (*r* = − 0.38, *P* = 0.092). In turn, changes in saturation and hue were strongly correlated (*r* = − 0.70, *P* < 0.001).

## Discussion

The findings in this study suggest that preen oil influences bib colouration in male house sparrows. This conclusion arises from two evidences. First, bib saturation was correlated with uropygial gland size—which is a good surrogate of preen oil production (Pap et al., 2010). Although several works have found an effect of preen oil on feather colouration (e.g., López-Rull, Pagán & García, 2010; Amat et al., 2011; Pérez-Rodríguez, Mougeot & Bortolotti, 2011), I am aware of only other work reporting a relationship between natural inter-individual variation in plumage colouration and uropygial gland size, concretely in great tits (*Parus major*), in which yellow brightness is positively correlated with uropygial gland size (Galván & Sanz, 2006). In addition, in house sparrows, uropygial gland size is positively correlated with the size of the wing bar (Moreno-Rueda, 2010). As a whole, these studies suggest that part of the inter-individual variation in bird plumage colouration is due to differences in the capacity of individual birds to produce preen oil.

Second, during the experiment, in control birds, changes in saturation of bib colouration correlated with changes in uropygial gland size. Uropygial gland size is determined as a result of the rate of preen oil production minus the rate of preen oil use in preening. Therefore, changes in uropygial gland size may be due to changes in preen oil production and/or changes in preen oil use in preening. At this point, and without behavioural observations, it is unknown what was the cause of changes in uropygial gland size, but in any case, the covariation between changes in uropygial gland size and changes in bib saturation suggests a link between preen oil and bib colouration. In other words, the findings in this experiment suggest that short-term changes in bib saturation were related to inter-individual differences in preen oil production or inter-individual differences in preen oil use in preening. It is well established that time invested in preening influences changes in colouration in birds (Zampiga, Hoi & Pilastro, 2004; Griggio, Hoi & Pilastro, 2010; Leitão & Mota, 2015), access to preen oil affects plumage colouration (López-Rull, Pagán, & García, 2010), and uropygial gland size correlates with plumage colouration (this study). Therefore, the consistent relationships between uropygial gland size and bib saturation—and the correlated changes in both variables—suggest that the (intra- and inter-individual) variation in bib saturation was related to the use of preen oil as a cosmetic, although the ultimate causal mechanisms remain to be elucidated.

However, given that the bib is black, one would expect changes in the achromatic part of the colouration, i.e., in the lightness. Strangely, lightness was the component of colouration least affected by the experiment, uropygial gland size, or change in uropygial gland size. In this sense, other studies have shown that preen oil reduces lightness (Delhey et al., 2008; Pérez-Rodríguez, Mougeot & Bortolotti, 2011). An examination of the a* (mean: 1.05; min–max: 0.40–2.24) and b* (mean: 1.56; min–max: −0.18–3.56) coordinates of hue, reveals that the bib hue is brown (black is not a hue, but the result of much reduced reflectance), and the change in uropygial gland size in control birds was related to a change in the saturation of that brown colour. A possibility is that the purity of colour (i.e., saturation) reflects how much the bib is free of dirt, and given that preen oil intervenes by cleaning plumage, the highest the preening effort the most saturated became the bib. It is also possible that the effect of uropygial gland on bib colour is mediated by bacteria: it has been shown that bacteria on plumage may alter feather colouration (Shawkey et al., 2007; Shawkey, Pillai & Hill, 2009; Gunderson, Forsyth & Swaddle, 2009; Leclaire et al., 2015), and preen oil may impact on plumage bacteria (Shawkey, Pillai & Hill, 2003; Reneerkens et al., 2008; but see Czirják et al. (2013) and Giraudeau et al. (2013)); therefore, preen oil might alter bacteria community or abundance on plumage, resulting in variation in colouration.

In addition to the results discussed above, bib colouration (hue and saturation) was correlated with body condition, and, indeed, changes in bib colouration during the experiment were also related to initial body condition. These findings imply that bib colouration—apparently partially dependent on preen oil- may indicate current physical condition. Therefore, the opportunity is opened for sparrows to update information contained in bib colouration by preening. Results in the experiment went in such a direction. Although the experiment failed to provoke detectable differences in preen oil production or use between experimental groups, it affected the way in which preening affected bib colouration changes during the experiment. That is, while in control birds the changes in uropygial gland size were related to changes in bib colouration, in LPS-treated birds, changes in uropygial gland size were unrelated to changes in bib colouration. The cause of this unexpected effect of the treatment is still unclear but, given that preen production or consumption seemed not to be affected by the experiment, the most plausible explanation is that the immune challenge affected preen oil composition. I hypothesize that immune challenged sparrows were precluded to synthetize the substances of preen oil that impact on bib colouration. This hypothesis would explain why in LPS-inoculated sparrows there was no relation between change in uropygial gland size and change in bib colouration, while in PBS-inoculated sparrows there was. If this hypothesis is right, it implies that preen oil composition of diseased birds would differ of that of healthy birds, and in this way they might show different colouration as well as odour, which is also implied in mate choice (e.g., Whittaker et al., 2013).

In any case, it should be stressed that the results in this study suggest that (1) preen oil seems modify bib saturation in “healthy” house sparrows, but (2) such an effect of preen oil is cancelled in immune challenged sparrows. Therefore, only healthy sparrows might modify bib saturation, and, in this way, bib colouration might have the potential to signal the current health of the bearer. Birds have been described to be able to signal their current health status by changing colouration of bare body parts, such as the beak, which quickly changes in colour in response to an immune activation (Blount et al., 2003; Faivre et al., 2003; McGraw & Ardia, 2003; Alonso-Alvarez et al., 2004). Also, plumage patches in some birds may vary their extension by tip abrasion (e.g., the black bib of house sparrows, Møller, Erritzøe, 1992). In this way, for example, pied flycatchers (*Ficedula hypoleuca*) indicate current health status by varying the size of their white forehead patch (Kilpimaa, Alatalo & Siitari, 2004). However, condition-dependent changes in feather colouration in completely moulted plumage has not been previously reported. Bear in mind that, although it is well documented that preen oil affects feather colouration (above), this is the first study showing a link between immune response, preen oil, and changes in plumage colouration in a bird species.

A still open question, nonetheless, is why the activation of the immune system would provoke changes in preen-oil composition or production. Preen oil is composed mainly of waxes, and therefore highly energetic components, undoubtedly very costly to produce, as implied by different lines of evidence. For example, food restriction in red knots (*Calidris alpina*) reduces their capacity to produce diester waxes, which are presumably more costly to produce than monoester waxes (Reneerkens, Piersma & Sinninghe Damsté, 2007). Moreover, experiments of immune activation conducted by Piault et al. (2008) and Moreno-Rueda (2015) suggest that preen-oil production is costly. Indeed, uropygial gland size has been found to be correlated with body condition and cell-mediated immune response in house sparrows (Moreno-Rueda, 2010). On the other hand, the activation of the immune system implies heavy energy costs (review in Schmid-Hempel, 2011; see Martin II, Scheuerlein & Wikelski (2003) for a study in house sparrows). Therefore, it is very likely that, in LPS-inoculated sparrows, the immune system and the uropygial gland competed for energy. Although it seems that preen-oil production was not impaired, it is possible that preen-oil composition changed to less energy-demanding waxes, as reported for red knots (see Reneerkens, Piersma & Sinninghe Damsté, 2007).

Lastly, it should be noticed that it is unknown whether the colour of the bib is used as a signal by house sparrows. Nevertheless, it presumably is used in this way, given that the signal depends on the eumelanin concentration (Jawor & Breitwisch, 2003). If this were not so, sparrows could cheat with a large but thinly melanised (lighter) bib but, by contrast, bib lightness is negatively correlated with bib size (Václav, 2006), suggesting that sparrows that may synthesise much eumelanin produce larger and darker bibs than do sparrows that synthesise less eumelanin. Moreover, the relationship found between bib saturation and body condition suggests that there is information contained in bib colouration. However, experimental studies modifying bib colouration would be welcome to test whether bib colour acts as signal.

In conclusion, the findings in this study suggest that healthy house sparrows modify bib colouration (in particular bib saturation) by preening. However, the effect of preen oil on bib colouration is cancelled in immune challenged sparrows. In this way, the results reported here suggest how house sparrows might use preening to update the information about their health status contained in the colouration of their bibs.

##  Supplemental Information

10.7717/peerj.2102/supp-1 Table S1 Raw data

10.7717/peerj.2102/supp-2 Table S2 Supplementary material

10.7717/peerj.2102/supp-3 Figure S1 Supplementary material

## Additional Information and Declarations

### Data Availability

The following information was supplied regarding data availability: Raw data are provided in Table S1.
