# Supplementary material for: Uropygial gland and bib colouration in the house sparrow
Source: PeerJ. 2016 Jun 2;4:e2102. doi: 10.7717/peerj.2102 (PMC4893339; doi:10.7717/peerj.2102)
Supplement: Table S1 [file peerj-04-2102-s001.docx]

**Table S1**

Raw data of the variables measured in this study. ID indicates the identification codex for each individual. Bib size is in cm^2^, mass is in g, wing length in mm. UGS is uropygial gland size, measured in mm^3^. L*, C* and H* are referred to lightness, saturation and hue, respectively.

| ID | Treatment | Bib size | Initial mass | Final mass | Wing length | Initial UGS | Final UGS | Initial L* | Initial C* | Initial H* | Final L* | Final C* | Final H* |
| --- | --- | --- | --- | --- | --- | --- | --- | --- | --- | --- | --- | --- | --- |
| 1M | LPS | 14.391 | 29.000 | 28.400 | 84.000 | 0.091 | 0.076 | 26.450 | 2.125 | 0.432 | 20.300 | 2.021 | 0.301 |
| BG-O | PBS | 13.716 | 27.600 | 26.500 | 81.500 | 0.148 | 0.075 | 11.440 | 0.866 | 1.024 | 16.370 | 1.942 | 0.436 |
| BN-O | LPS | 11.437 | 25.300 | 24.200 | 81.000 | 0.076 | 0.067 | 21.260 | 2.449 | 0.540 | 21.630 | 1.679 | 0.722 |
| BO-WW | LPS | 14.745 | 28.000 | 27.300 | 81.000 | 0.099 | 0.099 | 22.910 | 1.236 | 0.329 | 21.620 | 2.138 | 0.445 |
| BY-O | LPS | 18.531 | 27.500 | 26.900 | 82.000 | 0.120 | 0.082 | 17.510 | 1.946 | 0.818 | 15.860 | 1.427 | 1.026 |
| GG-O | LPS | 16.78 | 25.000 | 23.700 | 79.000 | 0.099 | 0.078 | 18.690 | 1.910 | 0.485 | 19.790 | 2.410 | 0.303 |
| GN-O | LPS | 15.868 | 27.400 | 26.300 | 80.500 | 0.137 | 0.114 | 21.290 | 1.524 | 0.664 | 17.430 | 2.635 | 0.486 |
| GO-WW | PBS | 19.224 | 29.100 | 27.900 | 80.000 | 0.075 | 0.064 | 17.040 | 1.408 | 0.537 | 35.080 | 3.156 | 0.060 |
| GP-O | PBS | 15.537 | 28.400 | 28.300 | 80.500 | 0.114 | 0.076 | 16.400 | 1.998 | 0.401 | 20.420 | 2.255 | 0.320 |
| GR-O | PBS | 15.955 | 27.900 | 27.800 | 82.000 | 0.114 | 0.060 | 15.420 | 1.046 | 1.744 | 25.750 | 1.329 | 0.609 |
| GW-WW | LPS | 12.978 | 22.800 | 23.400 | 76.000 | 0.072 | 0.059 | 19.440 | 3.154 | 0.790 | 21.730 | 3.873 | 0.466 |
| NG-O | PBS | 26.834 | 28.300 | 28.200 | 80.000 | 0.089 | 0.082 | 22.770 | 2.675 | 0.482 | 18.390 | 2.125 | 0.611 |
| NW-O | LPS | 15.4 | 25.400 | 25.600 | 81.000 | 0.087 | 0.085 | 22.710 | 3.298 | 0.330 | 20.850 | 3.444 | 0.409 |
| OR-WW | PBS | 23.446 | 28.800 | 28.100 | 80.500 | 0.129 | 0.103 | 18.650 | 0.966 | 1.457 | 20.110 | 1.529 | 0.566 |
| PB-O | LPS | 18.424 | 25.500 | 25.100 | 79.500 | 0.084 | 0.083 | 22.790 | 2.802 | 0.342 | 25.920 | 2.548 | 0.544 |
| PW-O | PBS | 15.459 | 26.000 | 26.900 | 82.000 | 0.131 | 0.107 | 15.140 | 1.607 | 1.071 | 18.163 | 1.572 | 0.855 |
| PY-O | LPS | 10.535 | 26.400 | 25.700 | 82.000 | 0.101 | 0.096 | 34.760 | 1.231 | 0.976 | 18.610 | 2.396 | 0.544 |
| WB-WW | LPS | 18.032 | 28.500 | 28.100 | 81.000 | 0.078 | 0.094 | 19.270 | 1.268 | 0.908 | 44.920 | 2.695 | 0.707 |
| WG-O | PBS | 20.455 | 26.900 | 26.300 | 80.000 | 0.113 | 0.133 | 28.290 | 2.996 | 0.354 | 28.780 | 1.280 | 0.758 |
| WN-O | PBS | 22.768 | 25.200 | 24.900 | 81.500 | 0.075 | 0.081 | 27.120 | 3.744 | 0.315 | 15.430 | 1.573 | 1.366 |
| WR-O | LPS | 17.911 | 24.300 | 24.000 | 80.000 | 0.102 | 0.123 | 14.107 | 2.682 | 0.174 | 18.140 | 2.706 | 0.383 |
