# Supplementary material for: Uropygial gland and bib colouration in the house sparrow
Source: PeerJ. 2016 Jun 2;4:e2102. doi: 10.7717/peerj.2102 (PMC4893339; doi:10.7717/peerj.2102)
Supplement: Table S2 [file peerj-04-2102-s002.docx]

**Table S2**

Results of the linear model examining the effect of treatment, change in uropygial gland size (ΔUGS), body condition and the interaction treatment×ΔUGS on bib lightness including an outlier detected in Figure S1.

|  | *β* ± SE | *F*_1, 16_ | *P* |
| --- | --- | --- | --- |
| Treatment | 183 ± 79 | 5.396 | 0.034 |
| Body condition | 0.47 ± 0.21 | 4.971 | 0.040 |
| ΔUGS | 0.24 ± 0.23 | 1.165 | 0.297 |
| Treatment*ΔUGS | -183 ± 79 | 5.398 | 0.034 |
