# Supplementary figures and images for: Uropygial gland and bib colouration in the house sparrow
Source: PeerJ. 2016 Jun 2;4:e2102. doi: 10.7717/peerj.2102 (PMC4893339; doi:10.7717/peerj.2102)

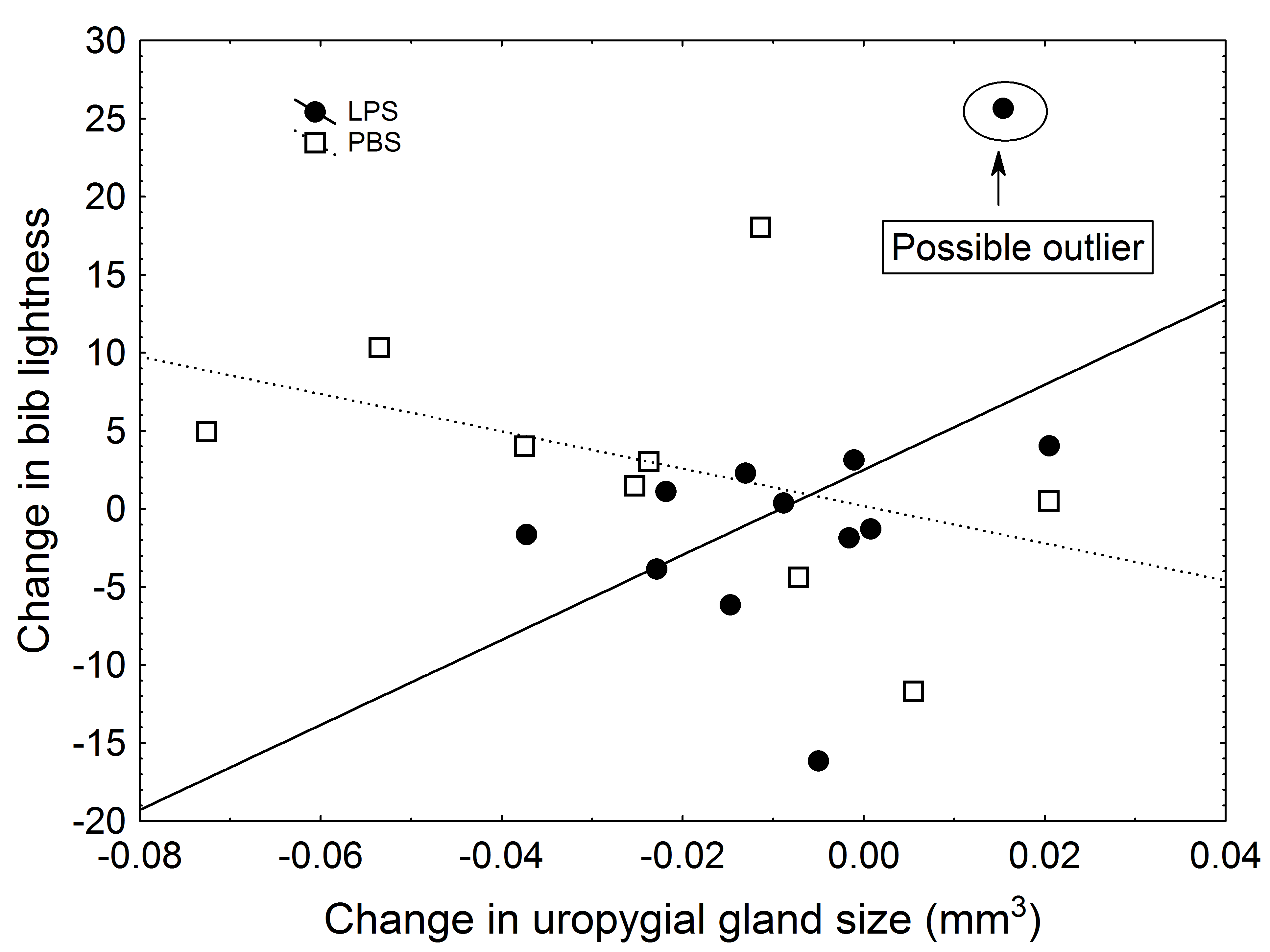

Supplement: Figure S1 [file peerj-04-2102-s003.png]
